# Gut Microbiota Dysbiosis in Depression: Pathological Correlations, Molecular Pathways, and Therapeutic Interventions

**DOI:** 10.3390/ijms27031530

**Published:** 2026-02-04

**Authors:** Jiaqi Cao, Jiayang Ma, Xu Zha, Xiaomei Bian, Wei Wang, Xicheng Liu

**Affiliations:** 1 Laboratory for Clinical Medicine, Department of Physiology and Pathophysiology, School of Basic Medical Sciences, Capital Medical University, Beijing 100069, China; cjq@mail.ccmu.edu.cn (J.C.);; 2 Laboratory for Clinical Medicine, School of Basic Medical Sciences, Capital Medical University, No. 10, Xitoutiao, Youanmenwai, Beijing 100069, China; majiayang@mail.ccmu.edu.cn

**Keywords:** major depressive disorder (MDD), gut microbiota (GM), gut–brain axis, probiotics

## Abstract

Major depressive disorder (MDD) ranks as a primary contributor to global ill health and disability, with treatments often proving insufficient. Recent study has increasingly found a strong correlation between gut microbiome diversity and mood-related behaviors, including MDD. Depression can alter gut microbiota (GM) composition, while intentional modulation of the GM may conversely influence depressive symptoms. This phenomenon arises from dynamic bidirectional interactions between the gut and brain, although the exact pathways are not yet fully elucidated. Proposed pathways include, but are not limited to, neural circuits, the endocrine system, immune responses, and metabolic regulation. Clinical data have also shown that regulating the GM through probiotics and prebiotics has the potential to alleviate depressive symptoms. This review summarizes contemporary research on the composition and modulatory functions of GM in MDD, and explores the predictive potential of GM for depression as well as the therapeutic prospects of probiotics, aiming to provide insights and directions for future research.

## 1. Introduction

Major Depressive Disorder (MDD), also termed as depression, is a widespread mental health disorder. It is marked by symptoms like low mood, reduced interest in activities, sluggish thinking, lack of motivation, self-reproach, disturbed eating and sleeping patterns, and in severe instances, suicidal ideation and actions [1]. Depression affects over 280 million people globally, with prevalence rising steadily each year. By 2030, its global financial burden is projected to nearly double due to medical costs, absenteeism, and related expenses. Despite the high prevalence of depression, recognition and treatment rates remain critically low, with only 9.5% of affected individuals receiving psychiatric services and a mere 0.5% obtaining adequate treatment [2]. Barriers include impaired self-awareness, social constraints, and the ease of symptom concealment in standard assessments [3]; moreover, even standard treatments fail in about 26% of cases [4], leading to high rates of non-response, recurrence, and increased risk of disability and suicide. Advancing the understanding of depression’s mechanisms and improving diagnostic and therapeutic strategies are therefore urgent scientific and clinical priorities.

The pathogenesis of depression involves complex biological, physiological, and social determinants, with potential for both independent occurrence and disease comorbidity [1,5]. Recent studies suggest that the hypothalamus–pituitary–adrenal (HPA) axis [1], inflammatory response [6], monoamine neurotransmitters, and neurotrophic factors (NTF) are critically involved in depression development and progression. Another pathway that has recently garnered widespread attention is the gut–brain axis [7].

The gut microbiota (GM), known as the “second brain [8,9],” is a complex ecosystem that significantly influences overall health [10]. Extensive research has established a clear link between GM dysregulation and mental illness, particularly depression [11,12,13,14,15,16]. Studies using germ-free (GF) and specific pathogen-free (SPF) animal models have demonstrated that GM composition affects stress responses and mood regulation [17,18,19,20]. Interventions such as fecal microbiota transplantation (FMT) [21], probiotics [22], and antibiotics [23,24] have been shown to markedly alter depressive-like behaviors in both animal models and human patients, underscoring the critical role of GM in depression.

Recent advancements in technologies like high-throughput sequencing, metabolomics, GF animal models, and bioinformatics have propelled research on the gut microbiota’s role in depression beyond the classical gut–brain axis framework into a more nuanced and dynamic phase. Emerging evidence reveals novel mechanisms, including microbial metabolites, bacteriophage–bacteria interactions, and immune cell subset regulation, and has spurred innovative interventions such as precision microbiota transplantation, next-generation probiotics, and engineered bacterial therapies. Yet, these rapidly evolving findings remain scattered and lack systematic integration into the literature. This review therefore synthesizes key developments from recent years, critically updating the latest mechanistic insights and emerging therapeutic strategies within the field of depression and the gut–brain axis.

## 2. Bidirectional Interactions Between GM and MDD in Both Directions

**Figure 1:**
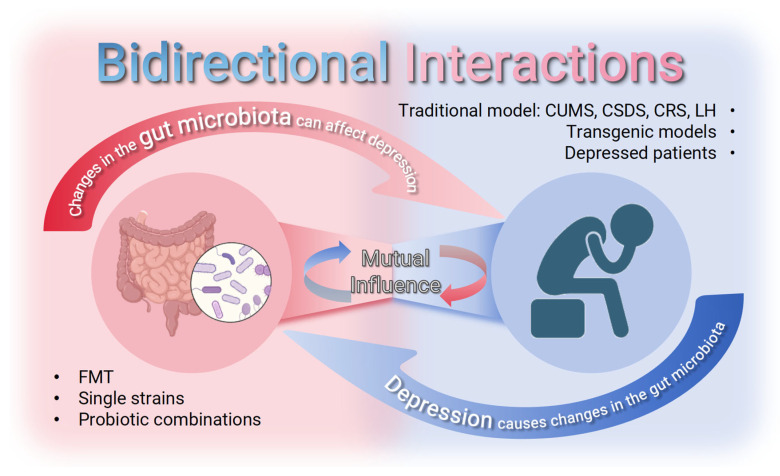
There is a two-way causal relationship between GM and depression: changes in GM occurred in depressed model animals and depressed patients; changes in the GM can affect depressive states.

A key challenge in GM and depression research is establishing their causal relationship, which is essential for uncovering mechanisms and developing treatments. Studies have compared microbial communities in depressed versus healthy subjects to assess depression’s effects on GM, while interventions such as FMT, antibiotics, and probiotics have been shown to directly modify depressive behaviors in animal models (Figure 1).

### 2.1. Changes in GM Occurred in Depressed Model Animals and Depressed Patients

Huangdi Neijing noted the psychosomatic link between emotions and the gut a long time ago [25]. Drossman later emphasized integrating psychological and physiological factors in diagnosing MDD [26]. Subsequent animal and clinical studies confirmed depression’s impact on the GM. Advances in sequencing technology revealed characteristic GM changes in depression, including reduced richness and diversity [14,27,28,29].

A common animal model of depression uses unpredictable chronic mild stress (CUMS), such as restraint, bright light, or shocks, to induce depression-like behaviors. Studies show CUMS reduces beneficial bacteria like *Lactobacillus* and *Bifidobacterium*, while increasing *Helicobacter* and *Proteobacteria* [30,31,32,33,34,35,36]. These GM changes are linked to impaired neurotransmitter production and hippocampal synaptic integrity [37,38]. Other models, including chronic social defeat stress (CSDS) [39,40], chronic restraint stress (CRS) [41], and learned helplessness (LH) [42,43], similarly show decreased microbial richness and specific taxonomic shifts, such as reduced *Firmicutes* or increased *Lactobacillaceae*. Maternal stress and depression also lower *Bifidobacterium* abundance in offspring, disrupting gut–brain axis regulation [44].

Genetically mediated factors contribute to 40–50% of depression cases, prompting the development of gene-edited animal models [45]. Studies show that knockout mice—such as *Chrna7* KO [46], *Gpr35* KO [47], *Negr1* KO [48], and *TGR5*-deficient [49] models—exhibit depression-like behaviors accompanied by distinct GM alterations. These include reduced beneficial genera such as *Dorea* and *Blautia*, increased taxa such as *Candidatus Arthromitus* and *Parabacteroides distasonis*, and GM dysbiosis. Specifically, host genetics can influence the gut environment through mechanisms such as mucosal immunity and neural signaling. For instance, *Chrna7* knockout may alter the microbiota via the subdiaphragmatic vagus nerve, thereby contributing to depression-like phenotypes [46]. Additionally, *Chrna7* deficiency can lead to splenic nerve hyperexcitation, triggering immune effects that result in thinning of the intestinal mucosal layer and subsequent changes in the gut microbial composition [49]. Similarly, decreased *Gpr35* expression induces alterations in the intestinal epithelial microenvironment, which in turn drives shifts in the GM [47]. Notably, supplementation with *Akkermansia* mitigated depressive phenotypes in *Negr1* KO mice [48]. Overall, these models consistently demonstrate significant and reproducible GM changes. These alterations have been reliably replicated across different animal models, highlighting their relevance in the context of depression (Table 1).

Clinical studies have consistently demonstrated characteristic GM alterations in individuals with depression, mirroring findings from animal models [51,52]. The two dominant phyla, *Firmicutes* and *Bacteroidetes*, which normally comprise over 90% of the human GM, show significant shifts in depression [53]. Decreased abundances of *Faecalibacterium*, *Roseburia*, *Bifidobacteriaceae*, and butyrate-producing taxa are negatively correlated with depressive symptoms [54,55,56]. Conversely, increased levels of *Bacteroidetes*, *Bacteroides*, *Parabacteroides*, *Proteobacteria*, and *Enterococcus* are positively associated with the disorder [57,58,59,60,61]. Microbial changes are more pronounced in severe cases, featuring elevated pro-inflammatory genera and reduced anti-inflammatory, short-chain fatty-acid-producing bacteria [62]. Butyrate synthesis impairment and disruptions in bacterial co-abundance networks further underscore the link between GM dysbiosis and depression [63].

Clinical studies have further identified distinct GM profiles associated with specific depressive subtypes and patient subgroups. Distinct gut microbial profiles at baseline distinguished patients who achieved remission from those who remained treatment-resistant after antidepressant therapy [64]. Comparative analysis revealed distinct microbiome configurations and gut–brain axis metabolic signatures between depressed adolescent and adult macaques. Specifically, CUMS-exposed adolescent cynomolgus monkeys showed disturbances in *Clostridium* and *Haemophilus* genera. In perinatal health, reduced microbial diversity and specific taxa such as *Candidatus Soleaferrea* are linked to higher risks of prenatal and postpartum depression [65,66,67,68,69]. Altered GM compositions are also observed in late-life depression [70], post-traumatic depression, and bipolar disorder-related depressive states [71,72,73]. Importantly, these microbial alterations appear to be more strongly associated with the psychiatric conditions themselves than with the use of psychotropic medications [74] (Table 2).

Taken together, the above findings suggest that stress and depressive states can lead to gut microbial dysbiosis. Caused by the differences in the racial strains and feeding environments of model animals, as well as the differences in the diet and living environment of depressed patients from different regions, many studies have not obtained completely consistent results on microbial population changes. But there is no doubt that a depressed mental state can affect the structural reorganization of gut microbial communities.

### 2.2. Alterations of GM Can Affect Depressive States

The most compelling causal evidence connecting GM to depression originates from FMT studies. Transplanting microbiota from depressed donors, whether from humans or animal models, into healthy rodents consistently induces depressive-like behaviors, including anhedonia and social avoidance [27,28,75,76]. Conversely, FMT from healthy donors ameliorates such behaviors, reduces inflammation, and elevates fecal short-chain fatty acids (SCFAs) in depressed models [77,78,79]. Experimental rodent models demonstrate that maternal depression can induce transgenerational behavioral phenotypes in offspring via gut microbiome-mediated mechanisms [80]. Preliminary clinical applications also suggest that FMT is a safe and feasible interventional strategy for depressive patients [81,82].

Following FMT research, studies on specific probiotic strains or combinations have shown significant antidepressant effects. *Lacticaseibacillus rhamnosus* and *L. helveticus* NS8 were found to alleviate depressive behaviors in rodents, sometimes outperforming conventional antidepressants [32,83,84,85,86,87]. *Bifidobacterium* supplementation enhanced stress resilience and reduced depressive symptoms [88,89]. *Akkermansia muciniphila* counteracted stress-induced behaviors and molecular changes [48,90]. Multi-strain probiotics, such as an 8-strain mixture and OttaBac^®^, also effectively reversed depression-like phenotypes in various models [91,92].

Collectively, these studies indicate that gut dysbiosis can result in impaired social behavior and heightened vulnerability to anxiety or depression. The administration of certain beneficial bacterial strains and probiotics may help restore balance to the dysregulated GM, thereby alleviating an individual’s depressive state or improving their mood (Table 3).

### 2.3. GM and Depression Interact with Each Other

Both of these directions are supported by a large amount of research data, and can at least be activated to achieve this. In fact, the GM–depression relationship exhibits bidirectional regulation, where microbial dysbiosis influences depressive phenotypes while depression concurrently alters gut microbial composition [93,94].

Rats subjected to social defeat stress exhibited a depressive phenotype and showed changes in their GM; the feces of the depressed model rats was given to the normal rats, and the normal rats showed a depressive phenotype [76]. Feeding rats with *Mycobacterium neoaurum*, which was isolated from the feces of individuals with depression, can also induce depressive-like behaviors [95]. The same related research shows that CUMS was used to induce depressive phenotypes in mice and their fecal FMT was given to young mice, and it was found that the young mice showed depressive changes at the cellular level and behavioral level. *Lactobacillus* supplementation mitigated the adverse effects of microbiota transfer from CUMS mice [96]. Chronic stress triggers concurrent GM dysbiosis and depressive behaviors, and *Streptococcus thermophilus* can reverse this depressive-like behavior [97]. The depressive phenotypes caused by psychological stress correlate with the levels of *Parabacteroides*, and colonization of *Parabacteroides distasonis* in wild-type mice can also induce a depressive phenotype [47]. Data analysis using the two-sample mendelian randomization (TSMR) also confirmed GM-MDD bidirectional causality. It identified 10 groups of GM associated with increased MDD risk and 10 groups that have a protective effect [57].

The two-way validation in multiple articles is enough to show that gut microbes and depression affect each other, but the mechanism has not been systematically studied, and the process of interaction between the two is still inconclusive. Moreover, the strong mechanistic evidence of causality obtained from animal models like GF mice faces challenges in ecological validity when translated to the complex human system, including the complexity of human microbiota, uncontrollable environmental factors, and the impact of comorbidities.

## 3. The Mechanism by Which the GM Acts on Depression

**Figure 2:**
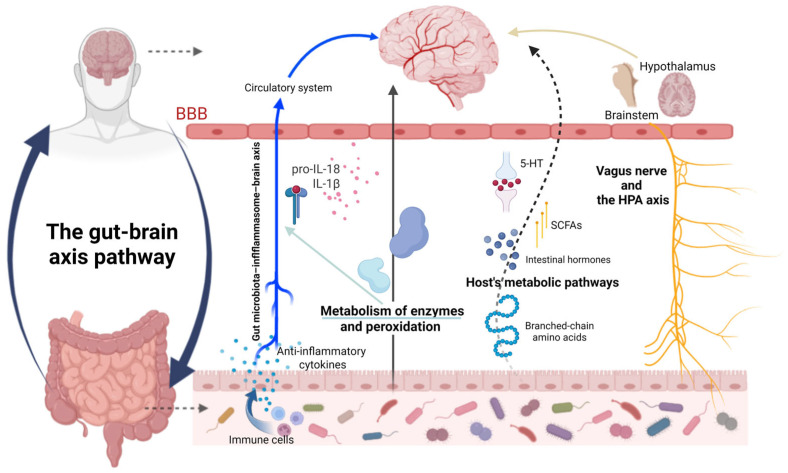
Multiple interconnected pathways mediate gut–brain axis regulation of depression pathogenesis and progression: including the vagus nerve and HPA axis; GM-inflammasome-brain axis; the host’s metabolic pathways; and the effects of enzyme metabolism and peroxidation. Created with BioRender (https://app.biorender.com/illustrations/66b5beb31c5373e670b8777f?slideId=dd0b40bd-7ff5-4946-8365-a24d4348ed08), accessed on 22 January 2026.

Alterations in GM are associated with emotional states, yet the mechanisms underlying their interaction remain unclear. The prevailing view is that their relationship is not a simple one-way cause-and-effect but rather a bidirectional, cyclical interaction that can form a vicious cycle. Consequently, mechanistic studies often employ bidirectional validation approaches. It is currently believed that the gut–brain axis facilitates communication through multiple pathways, including the nervous, immune, and endocrine systems, as well as metabolic processes (Figure 2). Critically, these pathways do not operate in isolation. For instance, microbial metabolites like SCFAs can exert anti-inflammatory effects (immune pathway) while also modulating neuroendocrine activity (HPA axis) and epithelial integrity. Similarly, vagal afferents (neural pathway) are sensitive to inflammatory cytokines and gut hormones. Therefore, the mechanisms outlined below represent dominant, yet highly interactive, channels in the gut–brain dialog, with the predominating specific pathway(s) likely depending on the nature of the dysbiosis and host context [98].

### 3.1. The GM Influences Depression Through the Vagus Nerve and the HPA Axis

The vagus nerve is a key pathway for gut–brain communication [99]. Studies show that subdiaphragmatic vagotomy prevents the development of depression-like behaviors in rodents following FMT from depressed donors. For example, *Lacticaseibacillus rhamnosus* loses its antidepressant effects after vagus nerve transection [22,100]. Similarly, transplanting microbiota from stressed mice did not induce depressive phenotypes in vagotomized recipients [46,101,102]. These results confirm that the subdiaphragmatic vagus nerve is essential for transmitting gut microbiota-driven depressive signals.

The HPA axis is another major pathway in gut–brain communication. Vagal afferents connect to hypothalamic structures, allowing stress-induced HPA activity to be modulated by gut signals [103]. Communication between the hypothalamus and gut through the HPA axis represents a fundamental element of brain–gut interactions, but recent studies suggest that communication on the HPA axis is bidirectional, with the gut also being capable of sending signals to the brain [104]. For instance, olfactory bulbectomized (OB) mice exhibited depression-like behaviors and prefrontal cortex impairment, accompanied by increased adrenocorticotropin-releasing hormone (CRH) levels and HPA hyperactivity [105]. These changes correlated with altered GM, suggesting that HPA activation influences colonic motility and microbial composition, which in turn may feed back to the brain.

### 3.2. The GM Influences Depression Through Inflammatory Pathways

Many studies suggest that the GM affects brain function via inflammatory signaling pathways [54,106,107,108], thereby influencing depression and anxiety-related phenotypes. In fact, the gut harbors a large number of immune cells. When pathogens or harmful substances breach the physical barrier of the intestinal epithelial mucus layer, they activate the gut immune cells. These activated immune cells not only produce pro-inflammatory cytokines to combat the threat but also subsequently release anti-inflammatory cytokines to regulate and terminate the inflammatory response, thereby preventing excessive tissue damage. This forms part of the intricate and finely tuned immune defense and regulatory network of the gut [109].

Inflammasome signaling, particularly involving caspase-1 and cytokines like IL-1β, plays a key role in mediating these effects [110]. Probiotic treatments, including multi-strain supplements and butyrate-producing bacteria, have been shown to alleviate depressive behaviors and reduce neuroinflammation [21,111,112]. Mechanistic studies have highlighted specific pathways that promote pro-inflammatory responses and synaptic pruning. One key pathway involves the upregulation of TRANK1, a robust risk gene for bipolar disorder. Notably, serum mRNA levels of TRANK1 are also elevated in patients with depression. Another pathway is microglial activation induced by LPS [36,113]. Consistently, human and animal studies associate depression with a shift toward pro-inflammatory microbial taxa and reduced SCFA producers [114]. Notably, the absence of Th17 cells confers resistance to microbiota-induced behavioral changes, underscoring the essential role of immune activation in gut–brain communication [115,116].

### 3.3. The GM Influences Depression via Host’s Metabolic Pathways

The GM produces neurotransmitters [117], SCFAs, branched-chain amino acids, and intestinal hormones, all of which may enter the host body through intestinal absorption [118,119,120]. Therefore, the alterations in host metabolic pathways caused by metabolites produced by the GM might be vital for gut–brain communication.

GM significantly influence 5-Hydroxytryptamine (5-HT) signaling, a key pathway in mood regulation [121]. *Clostridium butyricum* elevates the 5-HT, GLP-1, and BDNF levels [122]; *Bifidobacteria* influences 5-HT and BDNF expression [123]; *Lactococcus lactis* E001-B-8 enhances 5-HT metabolism [124]; *Lactobacillus reuteri 3* promotes 5-HT biosynthesis while inhibiting its degradation via the kynurenine pathway [125]; and *Akkermansia* normalizes 5-HT metabolism in both the brain and the gut [126]. Beyond single strains, 13 bacterial genera linked to depression are involved in synthesizing neurotransmitters such as γ-aminobutyric acid (GABA), glutamate, butyrate, and homovanillic acid, highlighting a broad microbial role in regulating neurochemical balance [37,59,97].

Amino acid metabolism is also one of the mechanisms of intestinal microbiota affecting depressive symptoms [127]. FMT from depressed patients into GF mice disrupts carbohydrate and amino acid metabolic pathways, coinciding with the emergence of depressive phenotypes [27]. In particular, altered tryptophan metabolism plays a central role: reduced tryptophan-derived indole metabolites are linked to depression-like behaviors, while supplementation with tryptophan, indole, or specific bacteria such as *Parabacteroides* and *Roseburia intestinalis* can restore metabolic balance and alleviate symptoms [41,47,128,129]. Similarly, elevated proline, both in circulation and diet, correlates with depression severity and induces depressive behaviors in mice, accompanied by changes in cerebral proline transporter expression [130]. These findings highlight microbial amino acid metabolism as a promising target for antidepressant interventions.

SCFAs, particularly propionate and butyrate, function as crucial gut–brain axis mediators. Once absorbed through the colonic mucosa, they enter systemic circulation to influence peripheral organs, including the brain. Functionally, SCFAs exist in protonated or deprotonated forms, with the latter requiring specific transporters expressed in tissues like the gut and brain. Altered SCFAs levels are consistently observed in depression: studies show reduced beneficial bacteria such as *Bifidobacteria* and *Butyricimonas*, which are key SCFAs producers, along with decreased SCFAs synthesis pathways in individuals with depressive symptoms [96,111,131,132]. Conversely, SCFAs supplementation alleviates anxiety- and depression-like behaviors in rodent models, restoring gut barrier integrity and metabolic balance [133,134]. Importantly, beyond these general pathways, certain SCFAs can directly modulate brain cell epigenetics. A key example is valerate (VA): produced by gut bacteria, VA enters circulation and inhibits histone deacetylases (HDACs) in the brain, exerting neuroprotective effects. This links bacteria involved in VA production and metabolism to depression, thereby bridging specific microbial metabolites with psychiatric symptoms through an epigenetic mechanism [135]. For instance, SCFAs counter methamphetamine-induced behavioral deficits via SIGMAR1 signaling, and compounds like Icariside II elevate SCFAs-producing genera, including *Akkermansia* and *Ligilactobacillus*, enhance tight junctions, and ameliorate depressive phenotypes [136,137]. These findings highlight SCFAs not only as metabolic and immune mediators but also as systemic epigenetic modulators, underscoring their potential as therapeutic targets or adjuvants in depression treatment.

The GM potentially influences depression development through the dysregulation of the peripheral and central nervous system glycerophospholipid and sphingolipid metabolism [138]. The antidepressant mechanism of escitalopram may involve the regulation of GM-dependent sphingolipid metabolic pathways. Alterations in gut microbial composition, particularly increased abundance of *Bacteroides pectinophilus*, showed strong correlations with elevated serum sphingolipid metabolites [139].

These metabolites and metabolic pathways improved depressive behavior and stress-induced changes in intestinal permeability in mice, but the specific regulatory pathways of metabolites acting on the brain are still unclear.

### 3.4. The Intestinal Microbiota Influences Depression Through the Metabolism of Enzymes and Energy Metabolism

Microbial 3β-hydroxysteroid dehydrogenase (3β-HSD) expression potentially contributes to depression pathogenesis through steroid hormone metabolism dysregulation. *Mycobacterium neoaurum* encodes 3β-HSD, which decreases testosterone levels in serum and the brain, then causes depressive-like behavior in mice [95]. Similarly, the GM can modulate estradiol levels by regulating synthetic enzymes or HPA axis function [140]. Additionally, 3β-HSD expressed by *Klebsiella aerogenes* TS2020 degrades estradiol in premenopausal women, and administering the bacteria back to wild mice results in depressive-like behavior in the mice [141].

GM-induced alterations in energy metabolism also contribute to the pathogenesis of depression. The microbial metabolite butyrate enhances mitochondrial biogenesis via AMPK-PGC1α pathway activation, while mitochondria-derived reactive oxygen species (ROS) reciprocally modulate GM composition through altering the intestinal epithelial microenvironment. Combinatorial therapies, such as probiotics, fiber supplementation, and FMT, can also reshape the microbiota–mitochondria crosstalk [142]. Mitochondrial dysfunction in intestinal epithelial cells alters GM composition, mediates oxidative stress, and transmits signals through the vagus nerve and enteric nervous system [143]. Oral administration of the antioxidant siSMAPo (TN) can reduce plasma ROS levels, improve depressive-like behavior in CRS mice, reduce pro-inflammatory cytokine levels, and protect gut barrier integrity [144].

At present, many studies have solved the mechanism of microbial antidepressant from different perspectives. However, to date, a comprehensive and systematic integration of these findings has yet to be achieved. Some researchers believe that these pathways do not operate independently, but rather form intricate regulatory networks that interact with numerous other molecules to achieve comprehensive regulation [145]. More notably, the choice of model may influence the conclusion. While supplementation with specific probiotics has demonstrated significant behavioral improvements and increasingly clarified mechanisms in animal models, their translation into human therapeutics requires cautious and stepwise validation. Therefore, we should look at these results with caution.

## 4. Application of Probiotics and Microbiota Modulation in Depression Treatment

**Figure 3:**
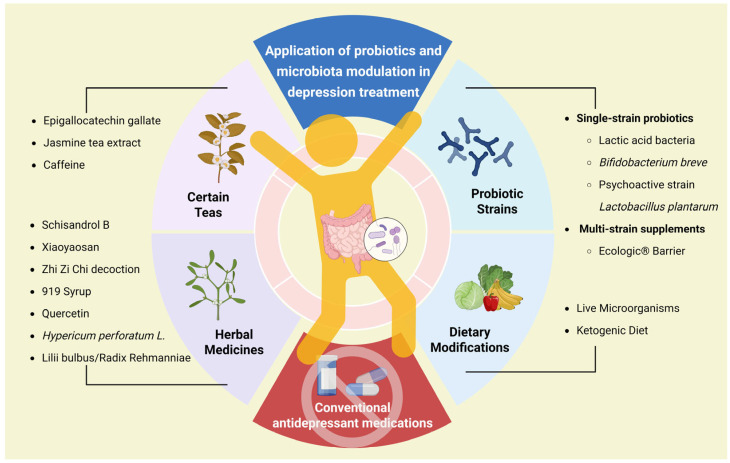
Traditional antidepressants are resisted by many patients, and modulation of the gut microbiota has become a more acceptable alternative, such as the use of single or mixed strains, certain teas, herbal remedies, and dietary modifications. Created with BioRender (https://app.biorender.com/illustrations/6874d8cfed55f74f67e4ec4f?slideId=21ec46c0-f985-4176-917d-3152590e46f7), accessed on 22 January 2026.

Conventional antidepressant medications carry significant side effects and risks of drug resistance, leading many patients to resist their use. In contrast, GM-based therapies offer a gentler and more acceptable alternative. Currently, multiple probiotic strains have been clinically applied, while certain teas, herbal medicines, and dietary modifications also alleviate depression through microbiota modulation (Figure 3).

### 4.1. Multiple Probiotics and Prebiotics Have Been Applied in the Treatment of Depression

Probiotics are viable microorganisms that confer health advantages when administered in sufficient doses. Accumulating clinical and preclinical evidence indicates that probiotic interventions exhibit antidepressant efficacy that is potentially comparable to conventional pharmacotherapies [146,147,148,149,150,151,152,153].

Clinically relevant probiotics, primarily lactic acid bacteria and bifidobacteria, demonstrate significant potential for alleviating depressive symptoms [91,154]. Key strains such as *Lacticaseibacillus casei*, *Lactobacillus helveticus*, *Bifidobacterium breve* CCFM1025, and psychoactive *Lactobacillus plantarum* have shown promising antidepressant effects in human studies [92,155,156]. Supplementation with these probiotics not only enhances microbial diversity but also specifically increases the abundance of Lactobacillus, which correlates strongly with reduced depressive symptoms [157].

Multi-strain probiotics demonstrate broader clinical efficacy compared to single-strain formulations in alleviating depression. Meta-analyses confirm that probiotic supplementation significantly reduces depressive symptoms across diverse populations [158,159], with enhanced benefits observed in individuals under 60 years old [160]. Adjunctive use with selective serotonin reuptake inhibitors (SSRIs) improves outcomes, even in treatment-resistant MDD, enhancing both mood and quality of life [161]. Probiotic interventions also correlate with increased abundance of beneficial bacteria such as *Akkermansia muciniphila*, which improved sleep quality, and modulation of 5-HT signaling [162,163]. Collectively, these findings suggest that probiotics can provide additional benefits as an adjunctive therapy, particularly in cases of drug-resistant depression where conventional medication alone is insufficient [164,165].

While probiotics show therapeutic potential for depression, certain studies report limited or inconsistent efficacy. Ecologic^®^ Barrier, a multi-strain probiotic, did not significantly improve depressive behaviors, cognition, or cortisol levels in rodent models [166]. Similarly, some human trials found no mood benefits in healthy individuals, though mild-to-moderately depressed patients showed improvement [167]. Additionally, depression recurrence after probiotic discontinuation has been observed, though long-term follow-up data are scarce, leaving relapse rates and underlying mechanisms unclear. These contrasting findings highlight the need for further research to clarify strain-specific effects, optimal treatment duration, and sustained efficacy. Moreover, critical questions regarding long-term safety and the ecological generalizability of single-strain interventions in diverse human gut environments require rigorous investigation in large-scale, longitudinal clinical trials [147]. The interaction between probiotics and conventional antidepressants also warrants careful study, as it may influence both clinical outcomes and the gut microbial ecology [168].

### 4.2. Traditional Herbal Medicines, Specific Foods, and Dietary Patterns Can Ameliorate Depressive Symptoms Through Modulation of the GM

Botanical compounds and traditional formulations demonstrate antidepressant potential through targeted modulation of the GM and associated metabolic pathways. Schisandrol B (SolB) reduces bile acid hydrolysis by inhibiting BSH-producing bacteria [169] and Xiaoyaosan (XYS) and Zhi Zi Chi decoction restore microbial balance and confer resilience in depression models, with effects transmissible via FMT [170,171]. Formulations like LBRD herbal formulation (Lilii bulbus/Radix Rehmanniae Recens co-decoction) and *Hypericum perforatum* L. (HPL) alleviate depressive phenotypes by suppressing neuroinflammatory pathways, such as the NF-κB/NLRP3 axis, and regulating key species such as *Akkermansia muciniphila* [172,173]. Also, 919 Syrup and quercetin modulate microbial composition, normalizing Firmicutes/Bacteroidetes ratio and tryptophan metabolism, while reducing hippocampal neuroinflammation [174,175].

Dietary components and nutritional patterns significantly influence depression through GM modulation. Bioactive compounds such as tea polyphenols [176], jasmine tea extract [177], and caffeine [178] alleviate depressive-like behaviors in animal models by reshaping GM composition, enhancing intestinal barrier integrity, and reducing pathogenic bacteria. Dietary habits are closely associated with the community structure of the GM [179,180]. Higher intake of dietary live microorganisms and adherence to gut-friendly diets correlate with reduced depression risk and suicidal ideation [181,182,183,184,185,186]. Specific diets, including the ketogenic diet, demonstrate antidepressant effects via GM-mediated mechanisms [187,188,189]. Moreover, dietary interventions show promise in mitigating depression comorbidities, such as in cancer patients [190].

## 5. Application of GM in the Diagnosis of Depression

Gut microbial signatures are emerging as potential diagnostic biomarkers for depression [191]. Multi-omics analyses reveal distinct microbial and metabolic profiles in patients, enabling the development of classifiers with moderate-to-good predictive accuracy (AUC up to 0.834). Characteristic changes include reduced *Escherichia* and *Shigella*, and increased *Coprococcus* and *Ruminococcus* [166,167,192]. Higher dietary microbiome-favorability scores also correlate with milder depression symptoms [193].

*Faecalibacterium prausnitzii*, a key commensal bacterium comprising 5–15% of the GM, exhibits strong anti-inflammatory and butyrate-producing properties, contributing to broad disease risk reduction [194]. Recognized as a promising next-generation probiotic, it is also proposed as a diagnostic biomarker for intestinal disorders [195]. Notably, its association with neuro-metabolic pathways supports its potential use as an auxiliary biomarker for depression [196].

However, significant caution is warranted. These performance metrics are often derived from single, retrospective cohorts and are susceptible to overfitting. The generalizability of such biomarkers across diverse populations with varying genetics, diets, and comorbidities remains largely unproven. Furthermore, population heterogeneity means that a single microbial signature may not be universally applicable. Prospective, multi-center validation studies are essential to determine the true clinical utility, specificity, and stability of microbiota-based diagnostic tools.

## 6. Deficiencies and Prospects

The gut microbiome significantly influences mood and behavior through multiple pathways, including the central nervous, immune, and metabolic systems. Some theories suggest that depressive behaviors may confer evolutionary advantages in certain contexts [197,198]. Despite the logical debate, elucidating the precise molecular and neural mechanisms underlying microbiota-mediated mood regulation remains a fundamental challenge in the field.

A major pragmatic challenge in microbiota research is limited reproducibility, which is largely due to methodological inconsistencies. Key technical variations in DNA extraction, sequencing platforms, and bioinformatics pipelines, introduce substantial bias [199,200]. For instance, the choice of extraction kit critically impacts results, as lysis efficiency varies for Gram-positive bacteria, altering perceived community structure and obscuring disease-associated lineages. Similarly, processing identical data with different bioinformatics pipelines can change diversity metrics and statistical significance [201]. A multi-lab comparison found that inter-lab technical variation could exceed biological variation between individuals [202]. Thus, a robust translation of findings demands greater standardization in protocols, consistent bioinformatics workflows, and cautious cross-study interpretation.

GM composition is shaped by diverse factors such as host genetics, diet, environment, experience, and sex differences, all of which must be considered in experimental design and interpretation [203,204,205,206,207,208]. Future clinical research must shift from proof-of-concept toward protocol optimization, rigorously investigating strain-specific dosing, treatment duration, and long-term safety. A critical priority is to elucidate interactions with conventional antidepressants, assessing impacts on both clinical efficacy and gut microbial ecology.

Beyond bacteria, emerging evidence highlights the roles of fungi, viruses, and microbiomes in other body sites, such as the oral and vaginal microbiomes, in mental health [167,209,210]. Advancing multi-omics technologies and integrated data analysis will be essential for deepening our understanding of microbiota–depression interactions and developing targeted preventive and therapeutic strategies [211].

## Figures and Tables

**Table 1:** Changes in GM in depressed model animals.

Subject	Sequencing Method	Main Findings	Ref.
CUMS C57BL/6J mice	16S rRNA sequencing	*Helicobacter*, *Bacteroides* and *Desulfovibrio* ↑ *Lactobacillus*, *Bifidobacterium* and *Akkermansia* ↓	[34]
CUMS C57BL/6J mice	Metagenomic sequencing	*Bifidobacterium longum*, *Roseburia intestinalis* ↓	[35]
CUMS C57BL/6J mice	16S rRNA sequencing	*Lactobacillus*, *Escherichia Shigella*, *Enterococcus* ↑	[36]
CUMS C57BL/6J mice	16S rRNA sequencing	*Escherichia, Shigella, Enterococcus, Vagococcus,* and *Aerococcus* ↑	[31]
CUMS C57BL/6J mice	16S rRNA sequencing	*Lactobacillus* ↓	[30]
CSDS C57BL/6 mice	16S rRNA sequencing	*Bifidobacterium* ↑ *Firmicutes* ↓	[40]
CRS C57BL/6J mice	16S rRNA sequencing	*Parabacteroides* ↓	[41]
LH SD rats	16S rRNA sequencing	*Lactobacillaceae* ↑	[42]
LH Wistar rats	16S rRNA sequencing	*Clostridiales incertae sedis* ↓	[43]
*Chrna7* KO C57BL/6 mice	16S rRNA sequencing	*Dorea*, *Blautia* ↓ *Candidatus Arthromitus* ↑	[46]
*Gpr35* KO C57BL/6J mice	16S rRNA sequencing	*Parabacteroides distasonis* ↑	[47]
*Negr1* KO C57BL/6J mice	16S rRNA sequencing	*Akkermansia* ↓	[48]
*TGR5* KO C57BL/6J mice	16S rRNA sequencing	*Anaeroplasma, Prevotella, Staphylococcus, Jeotgalicoccus,* and *Helicobacter* ↑ *Bifidobacterium* ↓	[50]

**Note:** ↑ indicates upregulation; ↓ indicates downregulation.

**Table 2:** Changes in GM in depressed patients.

Subject	Sequencing Method	Main Findings	Ref.
MDD patients	TSMR analysis	*Clostridiales* and *Parasutterella* ↓ *Oxalobacteraceae, Deltaproteobacteria,* and *Desulfovibrionales* ↑	[27]
MDD patients	16S rRNA sequencing	*Eggerthella, Holdemania, Gelria, Turicibacter, Paraprevotella* and *Anaerofilum* ↑ *Prevotella* and *Dialister* ↓	[14]
MDD patients	16S rRNA sequencing	*Faecalibacterium*, *Bacteroides*, *Roseburia*, *Parabacteroides* ↓	[54]
MDD patients	TSMR analysis	*Bacteroidetes*, *Parabacteroides* and *Bacteroides* ↑	[55]
MDD patients	High-throughput pyrosequencing	*Bacteroides*, *Proteobacteria*, *Actinomycetes* ↑ *Firmicutes*, *Faecalibacterium prausnitzii* ↓	[56]
MDD patients	16S rRNA sequencing	*Lachnospiraceae* ↓	[57]
MDD patients	16S rRNA sequencing	*Enterococcus* ↑	[58]
MDD patients	16S rRNA sequencing	*Sellimonas*, *Eggerthella*, *Lachnoclostridium*, *Hungatella* ↑ *Parvimonas micra*, *Coprococcus*, *Ruminococcus* (*UCG002*, *UCG003*, and *UCG005*), *Lachnospira UCG001*, *Eubacterium ventriosum*, *Ruminococcus gauvreauiigroup*, *Ruminococcaceae* ↓	[59]
MDD patients	Metagenomic sequencing	*Bifidobacterium longum* and *Roseburia intestinalis* ↓	[37]
MDD patients	16S rRNA sequencing	*Bifidobacterium*, *Blautia*, *Haemophilus* ↑ *Bacteroides*, *Faecalibacterium*, *Roseburia*, *Dialister*, *Sutterella*, *Parabacteroides*, *Bordetella*, *Phascolarctobacterium* ↓	[60]
Perinatal depression patients	16S rRNA sequencing	*Anaerostipes*, *Lachnospiraceae_UCG-001* ↑	[65]
Depressed pregnant women	Metagenomic sequencing	*Oscillibacter* sp. *KLE 1745* ↑	[66]
Prenatal depression women	16S rRNA sequencing	*Candidatus Soleaferrea* ↓	[67]
PPD patients	Genome-wide association study	*Ruminococcaceae UCG011, Veillonellaceae,* class *Clostridia* ↓ *Alphaproteobacteria, Slackia* ↑	[69]
Late-life depression patients	16S rRNA sequencing	*Enterobacter*, *Akkermansiaceae*, *Hemophilus*, *Burkholderia*, *Rothia* ↑	[70]
BD II depression	16S rRNA sequencing	*Bacteroides*, *Parabacteroides* ↑	[71]
Any depressive disorder	Metagenomic sequencing	*R. Bromii, Oscillibacter* ↓	[74]

**Note:** ↑ indicates upregulation; ↓ indicates downregulation.

**Table 3:** Changes in the GM can affect depressive states.

Subject	Treatment(s)	Outcomes	Ref.
Abx C57BL6/J mice	FMT from CORT-treated mice	Transferred both depressive-like phenotypes and associated gut microbial alterations	[76]
MDD patients	FMT from donor	Led to improvements in gastrointestinal symptoms and quality of life	[81]
PPD model mice	FMT from healthy donors	Alleviated depressive-like behaviors and inflammation	[78]
GF Kunming mice	FMT from MDD patients	Demonstrated significant social avoidance and depression-like behaviors	[27]
SD rats	FMT from MDD patients	Exhibited anhedonia and depression-like behaviors	[28]
SD rats	FMT from LL/resilient rats	Exhibited depression-like behaviors	[77]
Acute restraint stress BALB/c mice	*Lactobacillus rhamnosus* JB-1™	Reduced immobility time and corticosterone stress	[83]
CUMS mice	*Lactobacillu*	Reversed depression-like behavior	[32]
CUMS SD rats	*L. helveticus* NS8	Improved depression-like behavior and cognitive function	[84]
CSDS C57BL/6 mice	*Bifidobacterium*	Improved the depressive symptoms and enhanced stress resistance	[88]
MS SD rats	*Bififi-dobacterium infantis* 35624	Improved the depressive state	[89]
CRS C57BL/6 mice	*Akkermansia muciniphila*	Improved depression-like behaviors and restored abnormal changes in depression-related molecules	[90]
HFD or CON SD rats	A probiotic panel	Improved depressive-like behavior	[91]

## Data Availability

No new data were created or analyzed in this study. Data sharing is not applicable to this article.
